# Cognitive Change Before Old Age (11 to 70) Predicts Cognitive Change During Old Age (70 to 82)

**DOI:** 10.1177/09567976221100264

**Published:** 2022-09-15

**Authors:** Federica P. Conte, Judith A. Okely, Olivia K. Hamilton, Janie Corley, Danielle Page, Paul Redmond, Adele M. Taylor, Tom C. Russ, Ian J. Deary, Simon R. Cox

**Affiliations:** 1 Department of Psychology, University of Milano-Bicocca; 2 Lothian Birth Cohorts Group, The University of Edinburgh; 3 Department of Psychology, The University of Edinburgh; 4 Alzheimer Scotland Dementia Research Centre, The University of Edinburgh; 5 Division of Psychiatry, Centre for Clinical Brain Sciences, The University of Edinburgh

**Keywords:** cognitive development, intelligence, individual differences, statistical analysis, cognitive ability

## Abstract

Identifying predictors of cognitive decline in old age helps us understand its mechanisms and identify those at greater risk. Here, we examined how cognitive change from ages 11 to 70 is associated with cognitive change at older ages (70 to 82 years) in the Lothian Birth Cohort 1936 longitudinal study (*N* = 1,091 at recruitment). Using latent-growth-curve models, we estimated rates of change from ages 70 to 82 in general cognitive ability (*g*) and in three cognitive domains: visuospatial, memory, and processing speed. We found that *g* accounted for 71.3% of interindividual change variance. Greater cognitive gain from ages 11 to 70 predicted slower decline in *g* over 12 subsequent years (β = 0.163, *p* = .001), independently of cognitive level in childhood and at age 70, and domain-specific change beyond *g*. These results contribute to the goal of identifying people at higher risk of age-related cognitive decline.

This work addressed individual differences in cognitive aging from a novel perspective. Rather than studying how differences in age-related cognitive decline are associated with other factors, we examine cognitive-change consistency across the life course. We and others have shown that level of cognitive ability ascertained in childhood relates strongly to level of cognitive ability in older age (Deary, 2014; Rönnlund et al., 2015; Schalke et al., 2013). Here, instead, we asked whether individual differences in earlier life-course cognitive trajectories (age 11–70 years) predict subsequent cognitive change from age 70 to 82—a life period which generally sees more rapid and clinically important shifts. Individual differences in cognitive aging probably reflect an accumulation of small influences from numerous factors (Corley et al., 2018), many of which are likely to be already present in early life and midlife (e.g., genetic factors, early-life cognitive ability, physical fitness, smoking). Therefore, it is essential to characterize the relationship between earlier-period and later-period cognitive trajectories across the life course.

Cognitive decline is among the most feared aspects of aging. It will affect more people as the world population ages: In many countries, the proportion of older adults is increasing (Rousson & Paccaud, 2010; United Nations DESA, 2015), and longer life expectancy is not always matched by prolonged health (Abbafati et al., 2020; Prince et al., 2015). Even nonpathological cognitive decline can affect daily life and activities: Reduced cognitive functioning is associated with lower quality of life, leading to loss of autonomy, illness, and death (Batty et al., 2016; Deary et al., 2009; Schaie et al., 2009; Yam et al., 2014). Thus, the clear personal, societal, and financial consequences of cognitive aging, even among the nonclinical majority, motivate urgent scientific investigation. Beginning approximately at age 70, the risk of cognitive decline increases substantially (Deary et al., 2009; Marmot et al., 2003; Salthouse, 2019), as does the risk of dementia (Berr et al., 2005; Jorm & Jolley, 1998; Santoni et al., 2015).

There is considerable interindividual variability within the general trend of cognitive aging (e.g., Schaie & Willis, 2010; Zaninotto et al., 2018). Understanding the nature, predictors, and mechanisms underlying individual differences is essential for tackling the disruptive effects of cognitive decline and designing paths to successful aging. In this context, when some cognitive changes begin in early adulthood (Salthouse, 2019; Schaie & Willis, 2010; Tucker-Drob, 2019), the timing of interventions becomes an especially complicated matter (Plassman et al., 2010). The accurate prediction of trajectories is critical because it improves understanding of potential mechanisms and identification of those at relatively higher risk (Brayne, 2007; Deary et al., 2009; Salthouse, 2019).

Longitudinal studies emphasize the need to distinguish cognitive change from cognitive level; they show that an individual’s cognitive level at any given age is, at best, weakly associated with their cognitive trajectory (Karlamangla et al., 2009; Tucker-Drob, 2019). Accordingly, factors related to peak cognitive level in adulthood do not necessarily have a comparable association with cognitive-decline rates (Corley et al., 2018; Lövdén et al., 2020; Marden et al., 2017; Ritchie et al., 2016; Rönnlund et al., 2017; Tucker-Drob, 2019). Research on cognitive aging correlates has tested genetic, sociodemographic, health, and lifestyle factors. Among the stronger predictors of steeper cognitive decline are sex (being male), lower physical fitness, and possession of the *APOE* ε4 allele, whereas other predictors (e.g., childhood IQ, education) exhibit weaker effects (Blondell et al., 2014; Plassman et al., 2010; Ritchie et al., 2017; Schaie & Willis, 2010; Tucker-Drob, 2019; Zaninotto et al., 2018).

We are unaware of research examining whether differences in cognitive change from childhood to later adulthood are predictive of the subsequent cognitive-decline gradient in older age. This is an important omission. If we knew that individual differences in cognitive change between, say, ages 11 and 70 were associated with cognitive changes from ages 70 to 82, we would have more confidence that addressing factors operating before older age could ameliorate cognitive decline in older age.

**Statement of Relevance**

Age-related cognitive decline is a significant threat to quality of life in older age. Its economic and social impact on society will increase together with the steadily rising life expectancy. How can we preserve cognitive health in older age? Researchers have made significant advances in identifying protective and risk factors. However, most studies focus on a limited age range, and cognitive-change mechanisms are not yet completely understood. This work took advantage of almost-life-spanning longitudinal data to test whether cognitive trajectories across childhood and adulthood can predict cognitive trajectories in older age. Our findings show that earlier change is associated with later change. Some factors related to individual differences in cognitive change might thus operate over much of the adult life course, and certainly before older age. This knowledge can help us identify individuals at higher risk of decline and understand the mechanisms and factors responsible.

Here, we tested the hypothesis that cognitive change in general and domain-specific abilities after 70 (i.e., visuospatial, memory, and processing speed) might be predicted by cognitive change up to age 70. We used longitudinal data spanning 71 years from the Lothian Birth Cohort 1936 (LBC1936).

## Method

### Participants

The LBC1936 is a longitudinal study of cognitive, brain, and general aging. Participants were all born in 1936, and most took a test of general mental ability, the Moray House Test No. 12 (MHT), at age 11 years, as part of the Scottish Mental Survey of 1947 (SMS; Scottish Council for Research in Education, 1949). Between 2004 and 2007 (i.e., at about age 70), 1,091 probable SMS participants living in the Lothian area joined in the first wave of follow-up testing to form the LBC1936. As of 2022, the LBC1936 participants have taken part in five assessment waves at approximately 3-year intervals from age 70 to age 82. A description of the types of data collected at each wave is given in Taylor et al. (2018).

At baseline (Wave 1), the LBC1936 sample consisted of 1,091 individuals (543 females, age: *M* = 69.58 years, *SD* = 0.83). Table 1 presents sample demographics for all waves. Participants for whom age-11 MHT scores in childhood were not available (*n* = 63) or whose scores deviated more than 3.5 *SD* from the sample mean (*n* = 6) were excluded from analyses involving cognitive change between the ages of 11 and 70. The study was approved by the Lothian Research Ethics Committee (LREC/2003/2/39; Wave 1), the Multi-Centre Research Ethics Committee for Scotland (MREC/01/0/56; Wave 1), and the Scotland A Research Ethics Committee (07/MRE00/58; Waves 2–5).

**Table 1.** Sample Characteristics by Wave

Characteristic	Wave 1	Wave 2	Wave 3	Wave 4	Wave 5
*N*	1,091	866	697	550	431
Sex					
Men	548	448	360	275	209
Women	543	418	337	275	222
Mean age in years (*SD*)	69.58 (0.83)	72.54 (0.71)	76.30 (0.68)	79.38 (0.62)	82.06 (0.47)
Mean father’s social class (*SD*)	2.91 (0.94)	2.92 (0.94)	2.89 (0.94)	2.88 (0.95)	2.86 (0.95)
Mean father’s education years (*SD*)	9.96 (2.24)	9.98 (2.27)	9.96 (2.28)	10 (2.35)	10.09 (2.36)
Mean social class (*SD*)	2.4 (0.91)	2.36 (0.92)	2.33 (0.93)	2.26 (0.92)	2.23 (0.91)
Mean years of education (*SD*)	10.74 (1.13)	10.79 (1.14)	10.8 (1.14)	10.87 (1.18)	10.9 (1.17)

### Measures

The MHT was completed by participants at age 11 years and age 70 years (Wave 1) in the present study. It was called a verbal-reasoning test, but its items assess a range of abilities, including word classification, reasoning, analogies, arithmetic, spatial reasoning, and following directions. The test provides a single general cognitive-ability score, with a maximum value of 76. The MHT score correlated at about .80 with the Stanford-Binet scale in a validation test conducted during the SMS (Deary, 2014; Scottish Council for Research in Education, 1949).

Cognitive ability from age 70 to 82 was assessed using a battery of 10 tests related to three cognitive domains, administered at each wave from 1 to 5. Three tasks evaluated visuospatial ability: Matrix Reasoning and Block Design from the Wechsler Adult Intelligence Scale III UK (WAIS-III UK; Wechsler, 1998a), and Spatial Span forward and backward (the sum score of the two was used in the analyses) from the Wechsler Memory Scale III UK (WMS-III UK; Wechsler, 1998b). Three tests from the WMS-III UK evaluated verbal memory: Verbal Paired Associates immediate and delayed, Logical Memory immediate and delayed (for these two tasks, total scores were the sum of scores in the two conditions), and Digit Span backward. Finally, speed of information processing was ascertained by the Symbol Search and Digit-Symbol Substitution tasks from the WAIS-III UK by a visual inspection time task (Deary et al., 2007), and by a four-choice reaction time task (Deary et al., 2001). In the analyses, reaction times were multiplied by −1, so that, for all tests, higher scores indicated better performance. For a detailed description of test characteristics and administration, see Deary et al. (2007).

### Socioeconomic indicators

Participants reported their own and their father’s principal occupation (prior to retirement), which were grouped into social-class categories scored from 1 to 5: professional, managerial, skilled nonmanual, skilled manual, and semiskilled/unskilled. For women, spouse occupation was considered if higher than their own. Father’s and own years of education were also reported (Table 1).

### Statistical analysis

**Fig. 1.**
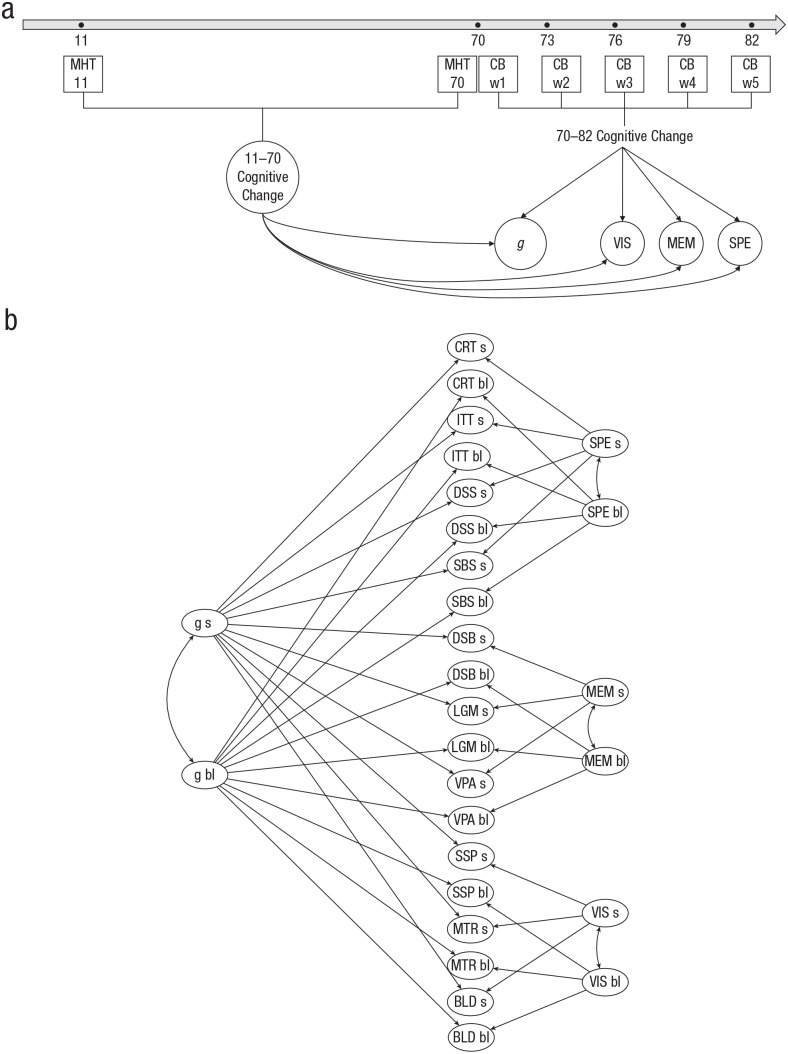
Analysis diagram and bifactor model representation. The diagram in (a) shows the main analysis: cognitive change from age 11 to 70 as a predictor of cognitive change from age 70 to 82. Cognitive change from age 11 to 70 was estimated from Moray House Test No. 12 (MHT) scores. Change in general cognitive ability (*g*) from age 70 to 82 and in visuospatial (VIS), verbal memory (MEM), and processing speed (SPE) domains were estimated from Cognitive Battery (CB) scores at Waves 1 through 5 (w1–w5). The bifactor measurement model of cognitive level and change is shown in (b). Factor-of-curves models (not illustrated) were used to derive baseline level (bl) and slope (s) parameters for each cognitive task. General cognitive ability (*g*) baseline level and slope (left) and domain-specific baseline level and slope (right) were extracted as second-level latent factors from task parameters (center). BLD = Block Design; MTR = Matrix Reasoning; SSP = Spatial Span; VPA = Verbal Paired Associates; LGM = Logical Memory; DSB = Digit Span backward; SBS = Symbol Search; DSS = Digit-Symbol Substitution; ITT = inspection time; CRT = four-choice reaction time.

We hypothesized that individual differences in cognitive change observed between the ages of 11 and 70 would be significantly associated with individual differences in cognitive change between the ages of 70 and 82. To test this hypothesis, we conducted the following steps, which are described in greater detail below: (a) estimate cognitive change from ages 11 to 70 using the MHT scores measured at both ages; (b) build measurement models for cognitive abilities from ages 70 to 82 using data from the larger set of 10 cognitive tests; (c) test the degree to which cognitive change between 11 and 70 predicts cognitive aging between 70 and 82; and (d) test whether cognitive change between 11 and 70 is independently predictive of change between 70 and 82, beyond just age-70 cognitive level (Fig. 1a).

#### Deriving measures of cognitive change

Cognitive change from 11 to 70 was modeled as the unstandardized residuals of the regression between MHT scores at Wave 1 (age 70) and age-adjusted MHT scores at age 11. This procedure has been used in previous Lothian Birth Cohort studies, such as Cherrie et al. (2018).

Cognitive change from age 70 to age 82 was estimated using a factor-of-curves (FOCUS) model (McArdle, 1988). At the lowest level of the FOCUS model, 10 linear latent growth curves estimated change for each of the 10 cognitive tests. Wave 1 (age 70) scores were considered the origins of the curves, and scores from subsequent waves (ages 73, 76, 79, 82) were weighted on the basis of the mean number of years that had passed since Wave 1. The latent growth curves provided, for each cognitive task, a baseline-level parameter, representing mean scores at Wave 1 (age 70), and a slope parameter, representing mean change per year for the subsequent 12 years.

At the higher level of the FOCUS model, baseline level and slope for each of the three cognitive domains (speed, memory, and visuospatial) and for *g* were estimated as second-order factors from cognitive tasks’ baseline levels and slopes. We fitted a bifactor structure: each task parameter loaded onto its domain factor and the general factor simultaneously. The general factor was constrained to be orthogonal to the cognitive-domain factors (Fig. 1b). Cognitive abilities are typically represented by hierarchical structures, with the most specific (i.e., individual task parameters) at the bottom and the most general (i.e., *g* parameters) at the top, separated by intermediate levels (i.e., domain parameters). This is also how LBC1936 data have been modeled in previous studies (Ritchie et al., 2016). In the present study, the bifactor model offered an advantage over the hierarchical model: It allowed common variance (*g*) to be partialed directly out of the cognitive-test scores and domains to be modeled as factors using variance from which *g* had been removed. Thus, we used the bifactor model to estimate the degree to which individual differences in cognitive-ability changes from ages 11 to 70 were associated with individual differences in *g* and orthogonal, domain-specific changes from ages 70 to 82. To repeat, any domain-related associations are independent of change that was common to all cognitive domains.

#### Estimating associations between cognitive change from ages 11 to 70 and ages 70 to 82

We asked whether our measure of cognitive change between ages 11 and 70 predicted subsequent cognitive declines in older age. To do so, we introduced change between ages 11 to 70 in the model of cognitive change from ages 70 to 82 (previously constructed; see above), as a predictor of the baseline (age 70) levels and slopes of general and domain-specific cognitive abilities within older age. To aid model convergence, we fixed factor loadings and intercepts obtained from the measurement model, whereas regression coefficients and residual-factor variances were freely estimated.

We introduced sex, the Sex × Cognitive-Change From 11 to 70 interaction, childhood and adult socioeconomic status indicators, and MHT score at age 11 as covariates alongside our main predictor. Following peer review, we ran follow-up analyses excluding either cognitive change between ages 11 and 70 or MHT score at age 11 from the regression model to test how the use of one versus both predictors influenced education associations with cognitive level and change.

Finally, we ascertained whether the measure of cognitive change between 11 and 70 accounted for unique variance in decline in *g* beyond baseline *g* between 70 and 82 years of age. We recognize that cognitive change between ages 11 and 70 would be correlated with the baseline level of cognitive functioning; as discussed above, the latter has previously been shown to correlate weakly with cognitive aging (Zaninotto et al., 2018). To examine the individual effects of the two measures (i.e., baseline level at age 70 and cognitive change at ages 11–70), we fitted a multiple regression model with general cognitive decline from 70 to 82 as a dependent variable and the FOCUS *g* baseline level and change between ages 11 and 70 as simultaneous predictors of slope. We included our covariates set.

#### Supplementary analyses

We calculated our main cognitive predictor (i.e., MHT change from ages 11–70) as a regression-based score because these are arguably less affected by random measurement error compared with raw difference scores (Campbell & Kenny, 2002; Cronbach & Furby, 1970). However, we recognize that there is no clear consensus on the optimal measurement of change. Therefore, we conducted a supplementary analysis in which we used a raw difference score, also accounting for change reliability (see Supplementary Methods in the Supplemental Material available online).

Even though the data benefited from a narrow age range, there were small age differences for each assessment wave in older age. To ensure that these age differences did not substantially impact our results, we fitted a second version of the cognitive measurement model, covarying the observed task scores with mean-centered age in days at the time of assessment.

Finally, in supplementary results, we present the association between cognitive change between 11 and 70 and individual cognitive domains, without partialing out general cognitive variance.

#### Peak-based measures of cognitive change

The longitudinal data from the LBC1936 cohort provides insight on cognitive change over most of the human life course. The lack of assessments between ages 11 and 70 makes it difficult to identify specific phases of cognitive change, such as childhood development or the beginning of decline in adulthood. However, we can use some existing data to partially fill the 60-year gap. One of the other measures collected in the LBC1936 is the National Adult Reading Test (NART; Nelson & Willison, 1991). The verbal skills assessed by the NART improve throughout adulthood and are robust to some normal and pathological decline (Lezak et al., 2004). Various follow-up studies of the SMS, using the MHT, have validated the NART as an estimate of prior or premorbid cognitive ability (Crawford et al., 2001; Deary & Brett, 2015; McGurn et al., 2008). Deary et al. (2004) showed that NART-included cognitive-change estimates correlate strongly with measures of actual lifetime cognitive change. As a counterpoint to our primary analysis, we used NART score at age 70 as an estimate of peak cognitive ability in adulthood. We then computed two additional regression-based indicators of cognitive change: from childhood to estimated adulthood peak (i.e., MHT at age 11 to NART at age 70); and from estimated adulthood peak to age 70 (i.e., NART at age 70 to MHT at age 70). The intention was to distinguish a phase of cognitive development from a phase of decline prior to age 70. Consistent with the main analysis, MHT score at age 11 was adjusted for age before regressing NART on it. Each of these indicators was tested as a predictor of cognitive change from 70 to 82 by introducing it in the cognitive-measurement models in the same way as cognitive change from 11 to 70.

#### Software, fit, and multiple-comparison correction

All models were estimated in the R environment (R Core Team, 2020) using the *lavaan* package (Rosseel, 2012) and a full-information maximum-likelihood algorithm, which capitalizes on information available from individuals even if they did not complete all assessments. We evaluated model fit on the basis of the root-mean-square error of approximation (RMSEA), standardized root-mean-square residual (SRMR), comparative fit index (CFI), and Tucker-Lewis indices (TLI): RMSEA lower than .05, SRMR lower than .08, and CFI and TLI larger than .95 indicate good model fit (Hu & Bentler, 1999). The resultant *p* values for the associations of interest were corrected for multiple comparisons with false discovery rate (FDR; Benjamini & Hochberg, 1995) using the p.adjust function from package Stats (R Core Team, 2020). Throughout the manuscript, we present standardized model estimates and the results marked as significant are those that survive FDR correction.

## Results

### Deriving measures of cognitive change

**Fig. 2.**
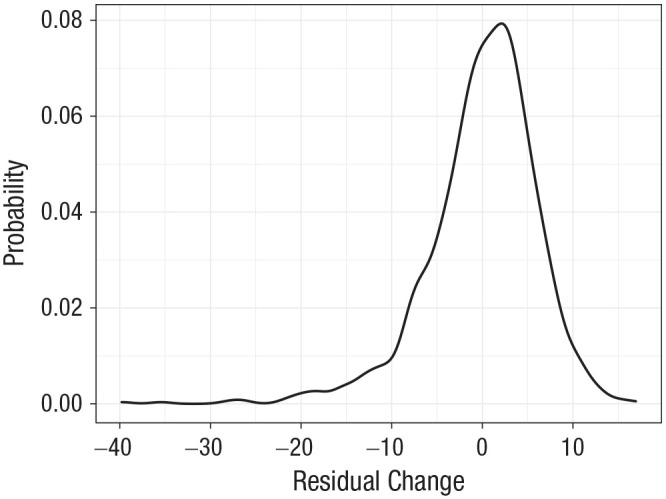
Density plot of residual-change scores of individual participants, showing residuals of the regression of Moray House Test scores at age 70 on age-corrected Moray House Test scores at age ~11.

MHT scores showed a general improvement between age 11 (*M* = 49.26, *SD* = 11.34) and age 70 (*M* = 64.23, *SD* = 8.80), with a mean increase of 15.23 points (*SD* = 8.36), on a maximum possible score of 76 (+0.26 points, or 0.02 *SD* units per year). Figure 2 illustrates the distribution of the age-adjusted regression residuals of MHT at age 70 on MHT at age 11 (interpreted as 11–70 cognitive-change measure in the analyses). Table 2 reports their correlations with the age-adjusted MHT difference score (see Section 2.3.3 in the Supplemental Material), with MHT at age 11 and 70, with *g* at age 70 (from the cognitive-measurement model), and with covariates. The regression-based measure of MHT change had a mean of 0.00 (*SD* = 6.09).

**Table 2.** Bivariate Correlations Between Measures of General Cognitive Ability and Cognitive Change From Ages 11–70

Variable	1	2	3	4	5	6	7	8
1. Residual change from ages 11–70								
2. Difference score from ages 11–70	.76***							
3. MHT at age 11	.00	−.65***						
4. MHT at age 70	.71***	.10**	.69***					
5. General cognitive ability (*g*) at 70	.39***	−.05	.53***	.64***				
6. Father’s social class	−.07*	.08*	−.21***	−.20***	−.17***			
7. Father’s education	−.03	−.08*	.09*	.05	.07*	−.37***		
8. Social class	−.12***	.16***	−.40***	−.35***	−.31***	.23***	−.18***	
9. Education	.13***	−.18***	.44***	.39***	.31***	−.34***	.33***	−.46***

Note: General cognitive ability (*g*) was estimated using a structural equation model including 10 cognitive tests. MHT = Moray House Test.

* *p* < .05. **p < .01. ***p < .001.

Fit indices for the bifactor model of the baseline levels and slopes of the 10 cognitive tests are presented in Table S1; factor loadings are presented in Table S2.

The cognitive-measurement model fit the data well. An average of 43% of task variance in baseline levels was shared within *g*, 22.3% was captured within domain, and 34.7% was task specific. On average, 71.3% of slope variance was captured by *g*, 19.6% was captured by domain factors, and 9.1% was task specific. The strongest indicators of *g* slope (i.e., of change rates in general cognitive ability) were the processing-speed tasks. Their loadings on the *g* slope factor ranged between 0.899 and 0.945, meaning that, on average, 85.5% of their slope variance was captured by *g.* This, in turn, resulted in little domain-specific slope variance: Only 7.5%, on average, was shared exclusively among processing-speed tasks (against 17.5% shared among visuospatial tasks and 37.8% among verbal-memory tasks).

### Cognitive change from ages 11 to 70 as a predictor of individual differences in later-life cognitive trajectories

**Fig. 3.**
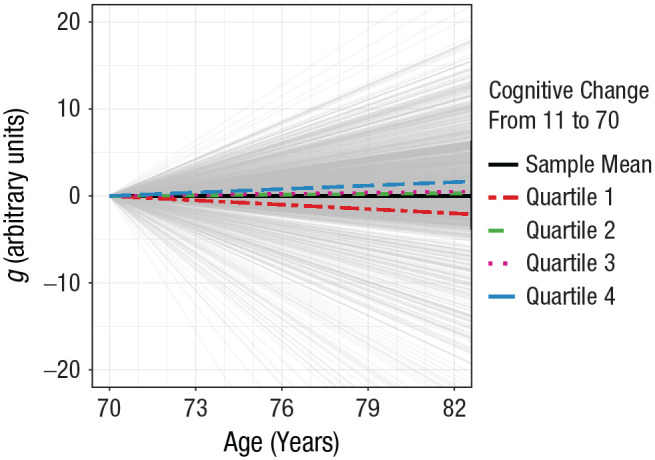
Estimates of standardized change in general cognitive ability (*g*) from age 70 to 82 by cognitive change between ages 11 and 70. Trajectories of standardized change per year are plotted over the course of 12 years. Individual change parameters were estimated from the bifactor cognitive-measurement model. Groups for cognitive change from ages 11 to 70 were identified through a quartile split of the sample.

Results of the principal analyses are presented in Table 3 (top). Table S1 reports model-fit indices, which were good. A greater relative improvement in MHT score between ages 11 and 70 was associated with slower decline in *g* from ages 70 to 82 (β = 0.156, *p* = .001): individuals who gained the most in MHT score up to age 70 tended to preserve their cognitive ability better from ages 70 to 82 (Fig. 3). A more marked improvement in MHT score was also associated with significantly higher *g* at age 70 (β = 0.356, *p* < .001). Childhood cognitive ability, measured by MHT score at age 11, predicted *g* baseline level (β = 0.456, *p* < .001) but not subsequent change. Participants’ years of education were unrelated to *g* parameters (β = 0.016, *p* = .436 for level, β = 0.014, *p* = .644 for slope), but follow-up analyses showed a significant positive association with *g* at age 70 when excluding either cognitive change from 11 to 70 or childhood cognitive ability from the predictors (β = 0.057, *p* = .010, and β = 0.119, *p* < .001, respectively). The associations between education and cognitive decline remained nonsignificant in these follow-up analyses (*p* ≥ .320).

**Table 3.** Associations Between Cognitive Change From Age 11 to 70 and Later-Life Trajectories of General and Domain-Specific Cognitive Abilities

Effect	Baseline level			Slope		
	β	95% CI	*p*	β	95% CI	*p*
General cognitive ability (*g*)						
Change from age 11 to 70	**0.356**	**[0.30, 0.41]**	**.000**	**0.156**	**[0.06, 0.25]**	**.001**
Sex	**−0.163**	**[−0.22, −0.11]**	**.000**	0.093	[0.01, 0.18]	.029
Change From Age 11 to 70 × Sex	0.050	[−0.01, 0.11]	.104	0.008	[−0.09, 0.10]	.863
MHT at age ~11	**0.456**	**[0.40, 0.51]**	**.000**	−0.022	[−0.12, 0.08]	.657
Father’s social class	0.006	[−0.05, 0.07]	.846	−0.034	[−0.12, 0.05]	.440
Father’s education	−0.020	[−0.09, 0.05]	.563	−0.023	[−0.12, 0.08]	.643
Social class	−0.030	[−0.09, 0.03]	.345	0.032	[−0.05, 0.12]	.470
Education	0.016	[−0.02, 0.06]	.436	0.014	[−0.05, 0.07]	.644
Visuospatial ability						
Change from age 11 to 70	−0.110	[−0.20, −0.02]	.017	−0.130	[−0.29, 0.03]	.110
Sex	−0.096	[−0.18, −0.01]	.029	0.103	[−0.04, 0.24]	.155
Change From Age 11 to 70 × Sex	−0.013	[−0.10, 0.08]	.780	**−0.237**	**[−0.39, −0.09]**	**.002**
MHT at age ~11	0.081	[−0.02, 0.18]	.112	**−0.455**	**[−0.60, −0.31]**	**.000**
Father’s social class	−0.021	[−0.11, 0.07]	.646	0.077	[−0.07, 0.22]	.299
Father’s education	0.060	[−0.04, 0.16]	.250	−0.047	[−0.22, 0.12]	.587
Social class	**−0.146**	**[−0.23, −0.06]**	**.001**	−0.004	[−0.15, 0.14]	.953
Education	0.019	[−0.04, 0.08]	.536	−0.000	[−0.10, 0.10]	.996
Verbal memory						
Change from age 11 to 70	0.037	[−0.05, 0.12]	.388	**0.155**	**[0.04, 0.27]**	**.010**
Sex	**0.346**	**[0.28, 0.42]**	**.000**	0.027	[−0.08, 0.13]	.616
Change From Age 11 to 70 × Sex	−0.055	[−0.14, 0.03]	.188	0.016	[−0.10, 0.13]	.789
MHT at age ~11	**0.215**	**[0.13, 0.31]**	**.000**	0.045	[−0.08, 0.17]	.469
Father’s social class	−0.045	[−0.13, 0.04]	.285	0.099	[−0.01, 0.20]	.067
Father’s education	−0.059	[−0.15, 0.04]	.225	0.057	[−0.06, 0.18]	.356
Social class	0.019	[−0.06, 0.10]	.658	−0.051	[−0.16, 0.06]	.351
Education	0.037	[−0.02, 0.09]	.198	−0.046	[−0.12, 0.03]	.209
Processing speed^a^						
Change from age 11 to 70	0.007	[−0.08, 0.10]	.878	−0.194	[−0.36, −0.03]	.022
Sex	**0.356**	**[0.28, 0.43]**	**.000**	−0.119	[−0.26, 0.02]	.105
Change From Age 11 to 70 × Sex	−0.094	[−0.18, −0.01]	.031	−0.006	[−0.17, 0.16]	.946
MHT at age ~11	0.009	[−0.09, 0.11]	.852	−0.012	[−0.19, 0.16]	.893
Father’s social class	−0.063	[−0.15, 0.02]	.150	0.100	[−0.05, 0.25]	.184
Father’s education	0.074	[−0.03, 0.17]	.147	0.163	[0.00, 0.33]	.057
Social class	−0.083	[−0.17, 0.00]	.060	0.013	[−0.14, 0.16]	.870
Education	0.008	[−0.05, 0.07]	.786	−0.110	[−0.21, −0.01]	.032

Note: Domain-specific cognitive abilities were measured in a bifactor model; domain-specific variance does not include variance common to all tasks (captured by *g*). The proportion of domain-specific slope variance beyond *g* is 17.5% (visuospatial), 37.8% (verbal memory), and 7.5% (processing speed). Boldface denotes significance (false-discovery-rate-corrected *q* < .05). CI = confidence interval; MHT = Moray House Test.

a The slope of the four-choice reaction time task loaded negatively on the domain factor.

MHT change between 11 and 70 remained a significant predictor of cognitive decline from 70 to 82 even after *g* at age 70 was entered as an independent variable in the multiple regression (ages 11–70 change: β = 0.163, *p* = .002; *g* baseline level: β = 0.132, *p* = .043). Cognitive trajectories from ages 11 to 70 thus appear more informative than cognitive functioning at age 11 or 70 in predicting subsequent cognitive-decline rates from age 70 to 82.

The next analyses involved changes in the cognitive domains from which variance in *g* had been removed (Table 3, middle and bottom). Note that there was about 3.5 times more slope variance in *g* than in the domains: The small amount of variance captured at the domain level warrants caution in interpreting the following results. More-favorable MHT cognitive trajectories at ages 11 to 70 were associated with slower decline in verbal memory (β = 0.155, *p* = .010) and with a steeper decline in visuospatial skills in women (Cognitive Change × Sex interaction: β = −0.237, *p* = .002). A nominally significant negative association was also present between processing speed and cognitive change at ages 11 to 70.

### Supplemental analyses

The Supplemental Material presents results from our analyses (a) measuring MHT change at ages 11 to 70 with a difference score, first on the entire sample and then on the subsamples showing reliable change in scores; (b) correcting for within-wave age differences; and (c) fitting individual domain models without partialing out general variance.

When conducting analyses on raw MHT change at ages 11 to 70, the direction and magnitude of effects on *g* level and change in the entire sample were consistent with those observed in the main analysis. The association with *g* slope was only nominally significant when assuming test-retest reliability of .90 for MHT scores and was not detected when assuming a reliability of .80. Raw change at ages 11 to 70 had a significant positive association with the slope of verbal memory in the full sample, but not with the other two domains or in the subsamples (Table S3).

Controlling for age differences within each wave of testing had no significant impact, as illustrated in Table S4.

The direction and size of effects in individual domain models (see Table S5 in the Supplemental Material) were essentially similar to those observed on *g* in the main analysis, reflecting the large proportion of variance shared across domains. The visuospatial-ability slope was the main exception, being significantly and negatively associated with MHT at age 11 but not with MHT change between ages 11 and 70.

### NART-based measures of cognitive change as predictors of individual differences in later-life cognitive trajectories

We found that MHT change from ages 11 to 70—that is, across nearly six decades—predicted subsequent cognitive changes from ages 70 to 82. We then used NART scores at age 70 as an estimate of peak adult cognitive ability to investigate whether change from childhood to peak ability, or from peak to age-70 ability, might be differentially important. We kept the same bifactor cognitive-measurement model as in the main analysis (see Section 2.3.1 in the Supplemental Material). Cognitive change from age 11 to peak was *M* = 0, *SD* = 5.82; it correlated with cognitive change from ages 11 to 70, *r* = .37, *p* < .001. Peak to cognitive change at age 70 was *M* = 0, *SD* = 6.59; it correlated with change from ages 11 to 70, *r* = .73, *p* < .001.

Having higher NART scores than expected on the basis of MHT at age 11 was associated with higher baseline level in *g* and domain-specific verbal memory (Table 4; βs = 0.122 and 0.210, respectively, *p* < .001). Having higher MHT scores at age 70 than expected on the basis of NART was associated with higher *g* baseline level (Table 5; β = 0.322, *p* < .001). However, cognitive change over shorter time spans, either between childhood and peak or between peak and age 70, appeared unable to predict *g* decline. In the Supplemental Material, we report model-fit indices and individual domain models for these analyses (see Tables S1 and S5).

**Table 4.** Associations Between Cognitive Change From Age-11 to Peak and Later-Life Trajectories of General and Domain-Specific Cognitive Abilities

Effect	Baseline level			Slope		
	β	95% CI	*p*	β	95% CI	*p*
General cognitive ability (*g*)						
11-NART Change	**0.122**	**[0.06, 0.19]**	**.000**	0.082	[−0.01, 0.17]	.083
Sex	**−0.214**	**[−0.27, −0.15]**	**.000**	0.077	[−0.01, 0.16]	.071
11-NART Change × Sex	0.021	[−0.04, 0.08]	.520	−0.025	[−0.11, 0.06]	.577
MHT at age ~11	**0.470**	**[0.41, 0.53]**	**.000**	−0.028	[−0.13, 0.07]	.585
Father’s social class	−0.003	[−0.07, 0.06]	.917	−0.032	[−0.12, 0.06]	.468
Father’s education	−0.052	[−0.12, 0.02]	.159	−0.032	[−0.13, 0.07]	.534
Social class	−0.051	[−0.12, 0.02]	.134	0.032	[−0.06, 0.12]	.481
Education	0.039	[0.00, 0.08]	.079	0.017	[−0.04, 0.08]	.582
Visuospatial ability						
11-NART change	−0.007	[−0.10, 0.09]	.886	−0.024	[−0.19, 0.14]	.771
Sex	−0.085	[−0.17, 0.00]	.052	0.096	[−0.05, 0.24]	.197
11-NART Change × Sex	0.021	[−0.07, 0.11]	.640	−0.155	[−0.30, −0.01]	.039
MHT at age ~11	0.092	[−0.01, 0.19]	.078	**−0.449**	**[−0.60, −0.30]**	**.000**
Father’s social class	−0.014	[−0.10, 0.08]	.768	0.087	[−0.07, 0.24]	.265
Father’s education	0.071	[−0.03, 0.17]	.178	−0.037	[−0.21, 0.14]	.681
Social class	**−0.136**	**[−0.23, −0.04]**	**.003**	0.009	[−0.15, 0.17]	.909
Education	0.009	[−0.05, 0.07]	.768	−0.010	[−0.12, 0.10]	.848
Verbal memory						
11-NART Change	**0.210**	**[0.13, 0.29]**	**.000**	0.092	[−0.02, 0.21]	.116
Sex	**0.332**	**[0.26, 0.40]**	**.000**	0.012	[−0.09, 0.12]	.818
11-NART Change × Sex	0.023	[−0.06, 0.10]	.570	0.018	[−0.09, 0.13]	.745
MHT at age ~11	**0.248**	**[0.16, 0.34]**	**.000**	0.039	[−0.09, 0.16]	.540
Father’s social class	−0.012	[−0.09, 0.07]	.780	0.102	[−0.01, 0.21]	.063
Father’s education	−0.047	[−0.14, 0.05]	.323	0.047	[−0.07, 0.17]	.449
Social class	0.061	[−0.02, 0.14]	.152	−0.045	[−0.16, 0.07]	.427
Education	0.009	[−0.05, 0.07]	.751	−0.043	[−0.12, 0.03]	.255
Processing speed^a^						
11-NART Change	−0.027	[−0.12, 0.06]	.559	−0.069	[−0.23, 0.10]	.413
Sex	**0.355**	**[0.28, 0.43]**	**.000**	−0.103	[−0.25, 0.04]	.166
11-NART Change × Sex	−0.023	[−0.11, 0.06]	.601	0.007	[−0.15, 0.16]	.928
MHT at age ~11	0.006	[−0.09, 0.10]	.903	0.001	[−0.18, 0.18]	.991
Father’s social class	−0.065	[−0.15, 0.02]	.143	0.107	[−0.04, 0.26]	.167
Father’s education	0.071	[−0.03, 0.17]	.167	0.185	[0.02, 0.35]	.032
Social class	−0.093	[−0.18, 0.00]	.040	0.016	[−0.14, 0.17]	.837
Education	0.014	[−0.05, 0.08]	.649	−0.125	[−0.23, −0.02]	.017

Note: Domain-specific cognitive abilities were measured in a bifactor model; domain-specific variance does not include variance common to all tasks (captured by *g*). The proportion of domain-specific slope variance beyond *g* is 17.5% (visuospatial), 37.8% (verbal memory), and 7.5% (processing speed). Boldface denotes significance (false-discovery-rate-corrected *q* < .05). CI = confidence interval; MHT = Moray House Test; NART = National Adult Reading Test.

a The slope of the four-choice reaction time task loaded negatively on the domain factor.

**Table 5.** Associations Between Cognitive Change From Peak to Age 70 and Later-Life Trajectories of General and Domain-Specific Cognitive Abilities

Effect	Baseline level			Slope		
	β	95% CI	*p*	β	95% CI	*p*
General cognitive ability (*g*)						
NART-70 Change	**0.322**	**[0.26, 0.38]**	**.000**	0.099	[0.00, 0.20]	.044
Sex	**−0.169**	**[−0.23, −0.11]**	**.000**	0.090	[0.01, 0.17]	.036
NART-70 Change × Sex	0.064	[0.00, 0.12]	.037	0.000	[−0.09, 0.09]	.996
MHT at age ~11	**0.325**	**[0.26, 0.39]**	**.000**	−0.072	[−0.17, 0.03]	.159
Father’s social class	−0.031	[−0.09, 0.03]	.312	−0.048	[−0.13, 0.04]	.273
Father’s education	−0.038	[−0.11, 0.03]	.275	−0.033	[−0.13, 0.07]	.514
Social class	**−0.084**	**[−0.15, −0.02]**	**.008**	0.009	[−0.08, 0.10]	.838
Education	**0.052**	**[0.01, 0.09]**	**.012**	0.029	[−0.03, 0.09]	.331
Visuospatial ability						
NART-70 change	−0.112	[−0.20, −0.02]	.017	−0.074	[−0.24, 0.10]	.391
Sex	−0.094	[−0.18, −0.01]	.031	0.094	[−0.05, 0.24]	.213
NART-70 Change × Sex	−0.053	[−0.14, 0.03]	.230	−0.058	[−0.22, 0.10]	.482
MHT at age ~11	0.129	[0.03, 0.23]	.015	**−0.422**	**[−0.58, −0.26]**	**.000**
Father’s social class	−0.011	[−0.10, 0.08]	.816	0.094	[−0.06, 0.25]	.221
Father’s education	0.064	[−0.04, 0.17]	.221	−0.044	[−0.22, 0.13]	.626
Social class	**−0.128**	**[−0.22, −0.04]**	**.004**	0.014	[−0.14, 0.17]	.852
Education	0.009	[−0.05, 0.07]	.767	−0.011	[−0.12, 0.09]	.829
Verbal memory						
NART-70 Change	−0.098	[−0.18, −0.01]	.025	0.104	[−0.02, 0.23]	.095
Sex	**0.331**	**[0.26, 0.40]**	**.000**	0.027	[−0.08, 0.13]	.618
NART-70 Change × Sex	−0.055	[−0.14, 0.03]	.183	−0.013	[−0.13, 0.10]	.830
MHT at age ~11	**0.246**	**[0.15, 0.34]**	**.000**	−0.006	[−0.13, 0.12]	.925
Father’s social class	−0.043	[−0.12, 0.04]	.303	0.087	[−0.02, 0.19]	.111
Father’s education	−0.068	[−0.16, 0.03]	.157	0.051	[−0.07, 0.17]	.409
Social class	0.016	[−0.07, 0.10]	.707	−0.072	[−0.18, 0.04]	.192
Education	0.042	[−0.01, 0.10]	.140	−0.034	[−0.11, 0.04]	.360
Processing speed^a^						
NART-70 Change	0.036	[−0.05, 0.13]	.436	−0.170	[−0.34, 0.00]	.044
Sex	**0.360**	**[0.29, 0.43]**	**.000**	−0.123	[−0.27, 0.02]	.095
NART-70 Change × Sex	−0.075	[−0.16, 0.01]	.078	0.033	[−0.13, 0.19]	.689
MHT NART	−0.001	[−0.10, 0.10]	.978	0.065	[−0.11, 0.24]	.474
Father’s social class	−0.064	[−0.15, 0.02]	.144	0.122	[−0.02, 0.27]	.103
Father’s education	0.072	[−0.03, 0.17]	.158	0.174	[0.01, 0.34]	.041
Social class	−0.087	[−0.17, 0.00]	.048	0.041	[−0.11, 0.19]	.591
Education	0.011	[−0.05, 0.07]	.719	**−0.130**	**[−0.23, −0.03]**	**.009**

Note: Domain-specific cognitive abilities were measured in a bifactor model; domain-specific variance does not include variance common to all tasks (captured by *g*). The proportion of domain-specific slope variance beyond *g* is 17.5% (visuospatial), 37.8% (verbal memory), and 7.5% (processing speed). Boldface denotes significance (false-discovery-rate-corrected *q* < .05). CI = confidence interval; NART = National Adult Reading Test.

a The slope of the four-choice reaction time task loaded negatively on the domain factor.

## Discussion

Our main finding is that individual differences in cognitive change between ages 11 and 70—measured on the same general-ability test—significantly predicted differences in *g* change from ages 70 to 82 in this narrow-age cohort. We are not aware of other studies comparing cognitive-change rates across these periods of life. The association we observed is modest but is at the upper bounds of typical effect sizes for individual risk and protective factors for cognitive aging in this cohort (e.g., Corley et al., 2018) and others (e.g., Zaninotto et al., 2018). Moreover, change between ages 11 to 70 was informative about decline rates even when we controlled for cognitive level in childhood and at age 70, thus offering independent predictive value. These findings fit an account of differential preservation (Salthouse et al., 1990), whereby individuals with similar cognitive levels decline at different rates depending on the amount of cognitive change experienced from youth to older adulthood. Our results encourage the search for cognitive-change determinants relatively early in the life course, as they are likely relevant to cognitive decline in later life.

We did not detect any significant association between years of education and cognitive level at age 70, in apparent contrast with previous LBC and meta-analytical investigations (Lövdén et al., 2020; Ritchie et al., 2016). However, according to follow-up analyses, the result is due to the simultaneous inclusion of childhood cognitive ability and cognitive change between 11 and 70 as model predictors, both of which correlate with education and consequently attenuate its associations with cognitive differences at age 70.

The bifactor model differentiated *g* variance (shared among all cognitive tasks) from variance specific to each cognitive domain. Compared with a previous study considering the first three assessments on the same cohort (Ritchie et al., 2016), investigating Waves 1 to 5 revealed a higher proportion of shared slope variance. This shift is consistent with Tucker-Drob and colleagues’ (2019) meta-analytic finding of dynamic dedifferentiation: *g* accounts for increasing amounts of variance with advancing age. Data from the present study and others (e.g., Tucker-Drob et al., 2019) shows that starting at about age 70, more than half of interindividual variability stems from differences in general, rather than from domain-specific cognitive decline. Therefore, accounting for *g* change should be a primary focus of cognitive-aging research.

The association of earlier cognitive change (ages 11–70) with later decline in *g* (ages 70–82) appeared robust in our study. Supplementary analyses showed that neither using an alternative measure of earlier cognitive change nor introducing age as an additional covariate changed this result appreciably.

The predictive effect of cognitive change from 11 to 70 seemed pervasive, being significant also with regard to domain-specific decline. Greater relative improvement in MHT scores from age 11 to 70 was associated with better preservation of verbal memory and with steeper decline in visuospatial abilities at later ages (the latter only in women). These effects were less stable than those on *g* (e.g., they did not survive FDR correction in the age-adjusted model); however, we note again the small amount of domain-specific variance compared with general variance. Altogether, our results support the initial hypothesis that changes between childhood and late adulthood might be relevant to a broad range of cognitive changes after age 70, especially concerning general cognitive ability. These are the first data suggesting that those with more positive earlier trajectories are at lower risk of subsequent decline into older age.

Why did change between 11 and 70 predict change in *g* better than in specific domains? First, in older age, there was much more variance in *g* change than in domain-specific changes. Second, the MHT test correlates strongly with the Stanford-Binet overall IQ score in childhood (Deary, 2014) and with *g* in adulthood (Deary et al., 2010). Therefore, it was likely to be good at predicting subsequent change in *g*. Performance in specific cognitive domains at ages 11 and 70 could have predicted domain-specific change better. We think it would be valuable if that could be tested for memory, which is a signature of some types of mild cognitive impairment and dementia.

New questions arise as to what lifetime period might be most informative about age-related cognitive decline: Would it be, say, between childhood and early adulthood, or from mid- to later life? We partially answered such questions using the NART at age 70 as an indicator of peak cognitive ability and assessing change in rank orders from age-11 MHT to NART and from NART to age-70 MHT. Previous Lothian Birth Cohort studies showed that NART-based cognitive-change estimates correlate strongly with actual cognitive change (Deary et al., 2004). Despite this, the absence of significant associations with rates of change in *g* suggests that neither childhood-peak change nor peak-to-age-70 change are in themselves sufficient to anticipate cognitive trajectories in older age. In this study, the longer time span (i.e., from ages 11 to 70) proved more informative about change rates in older age. However, additional research and alternative measures of cognitive change over shorter intervals (i.e., childhood to early adulthood, early to late adulthood) are needed to determine the relationship of cognitive-change trajectories.

### Limitations

Limitations should be considered when interpreting our results and may help inform future research. The Lothian Birth Cohort studies provide direct measures of participants’ cognitive abilities in childhood and older age. However, only a single MHT total score is available at each time point, preventing the testing of factorial invariance or latent-change-score modeling. Despite this, existing literature (e.g., Terman & Merrill, 1937) and consistency of results between main and supplementary analyses support our estimate of MHT test-retest reliability.

No cognitive tests were administered between ages 11 and 70. Thus, our measure of cognitive change likely reflects the lifelong influence of multiple factors and encompasses nonlinear changes (e.g., cognitive development and early decline). However, the effects of cognitive change between 11 and 70 were robust to the introduction of cognitive and socioeconomic covariates, and we could use a strong index such as the NART to further specify cognitive trajectories. We hope that further research can identify potential critical periods during which earlier-life cognitive change anticipates later-life decline.

Compared with the population average, LBC1936 cohort members tend to be healthier and better educated and tend to perform better on cognitive-ability tests (Taylor et al., 2018), likely leading to some restriction of range and a slight reduction in effect sizes (e.g., Johnson et al., 2011). Finally, participants were all born in a single year and come from a particular geographical setting, thereby limiting our results’ generalizability, albeit removing the possibility of cohort effects in a mixed-age sample.

### Conclusion

Research indicates that individual differences in cognitive decline arise from many diverse factors, each exercising a small influence (Corley et al., 2018; Deary et al., 2012). Tracing cognitive trajectories back through the life course requires data that are rarely available. The present study shows that cognitive change between ages 11 and 70 is independently informative of cognitive trajectories from ages 70 to 82, beyond cognitive level at age 11 or 70. Therefore, the results support identifying individuals at higher risk of cognitive decline before the critical years in which dementia risk accelerates. The positive side to the findings is that, to some extent, those who fare better cognitively from ages 11 to 70 tend to be at lower risk of cognitive decline from 70 to 82. To quote a sentence often attributed to Fred Astaire (1899–1987), “Old age is like everything else . . . to make a success of it, you’ve got to start young.”

## Supplemental Material

sj-docx-1-pss-10.1177_09567976221100264 – Supplemental material for Cognitive Change Before Old Age (11 to 70) Predicts Cognitive Change During Old Age (70 to 82) Click here for additional data file. Supplemental material, sj-docx-1-pss-10.1177_09567976221100264 for Cognitive Change Before Old Age (11 to 70) Predicts Cognitive Change During Old Age (70 to 82) by Federica P. Conte, Judith A. Okely, Olivia K. Hamilton, Janie Corley, Danielle Page, Paul Redmond, Adele M. Taylor, Tom C. Russ, Ian J. Deary and Simon R. Cox in Psychological Science

## Transparency

*Action Editor:* Karen Rodrigue

*Editor:* Patricia J. Bauer

*Author Contributions*

F. P. Conte, I. J. Deary, J. Corley, J. A. Okely, O. K. Hamilton, and S. R. Cox contributed to the conceptualization and methodological design of the study. In addition, F. P. Conte was responsible for the formal analysis and writing of the original draft; A. M. Taylor was involved in the investigation and project administration; I. J. Deary and S. R. Cox were responsible for project administration, funding acquisition, and supervision. D. Page contributed to investigation and data curation. P. Redmond contributed to data curation. All authors reviewed and edited the original draft and approved the final version for submission.

**Open Practices:** The data for this study can be requested by completing a data-request form from the Lothian Birth Cohort and are subject to the terms of a standard data-transfer agreement (https://www.ed.ac.uk/lothian-birth-cohorts/data-access-collaboration). Code is available at https://www.ed.ac.uk/lothian-birth-cohorts/summary-data-resources. The design and analysis plans for the present study were not preregistered.
