# Supplementary material for: Cognitive Change Before Old Age (11 to 70) Predicts Cognitive Change During Old Age (70 to 82)
Source: Psychol Sci. 2022 Sep 15;33(11):1803–17. doi: 10.1177/09567976221100264 (PMC9660354; doi:10.1177/09567976221100264)
Supplement: sj-docx-1-pss-10.1177_09567976221100264 – Supplemental material for Cognitive Change Before Old Age (11 to 70) Predicts Cognitive Change During Old Age (70 to 82) [file sj-docx-1-pss-10.1177_09567976221100264.docx]

**Supplemental Online Materials - Reviewed for:**

**Cognitive change before old age (11 to 70) predicts cognitive change during old age (70 to 82)**

Federica Conte^*^, Judith A. Okely, Olivia Hamilton, Janie Corley, Danielle Page, Paul Redmond, Adele M. Taylor, Tom C. Russ, Ian J. Deary, Simon R. Cox^*^

*To whom correspondence should be addressed:

[federica.conte@unimib.it](mailto:federica.conte@unimib.it)

Simon.cox@ed.ac.uk

Summary

[Supplementary methods 3](#_Toc94540113)

[Raw difference score for cognitive change from 11 to 70 years 3](#_Toc94540114)

[Individual domain models 3](#_Toc94540115)

[Supplementary results and tables 4](#_Toc94540116)

[Model fit indices 4](#_Toc94540117)

[Cognitive measurement model - factor loadings 6](#_Toc94540118)

[Estimating cognitive change from 11 to 70 years as a raw difference score 7](#_Toc94540119)

[Age-adjusted models 11](#_Toc94540120)

[Individual domain models 12](#_Toc94540121)

# Supplementary methods

## Raw difference score for cognitive change from 11 to 70 years

Raw cognitive change from age 11 to 70 was computed as the difference between MHT scores at Wave 1 (~70 years) and at the SMS1947 (~11 years). Change score reliability was estimated using the method detailed in Johnson, Gow, Corley et al. (2012). A Reliable Change Index (RCI) was computed as the ratio of raw change between two time points (x_1_ and x_2_) to its standard error (SE):

$$RCI=\frac{(x_{2}-x_{1})}{{SE}_{diff}}$$

The standard error of the difference (${SE}_{diff})$ was computed as follows:

$${SE}_{diff}= \sqrt{2{SE}_{m}^{2}}= \sqrt{2{SD}_{1}^{2}(1-R_{xx})}$$

$${SE}_{m}={SD}_{1}\sqrt{\left( 1-R_{xx} \right)}$$

where R_xx_ is the test-retest reliability. There is no published period-free reliability coefficient for the MHT instrument, so, based on its psychometric properties and correlation with the validated Stanford-Binet scale, we used an approximate value of .90 to indicate good reliability and, separately, an approximate value of .80 for a more conservative model.

## Individual domain models

Separate cognitive measurement models were estimated for each of the three cognitive domains assessed, without any superordinate (i.e., general cognitive ability) factor. Each model included only data from the cognitive tasks relevant to the domain in question. LGCs were used to estimate task baseline level and slope parameters, and domain parameters were estimated as second-order factors. Cognitive change from age 11 to 70 (or NART-based measures of cognitive change where appropriate), sex, cognitive change × sex interaction, and socioeconomic indicators were introduced as predictors of domain baseline level and slope.

Individual domain models were fit for each supplementary version of the main analysis, and results are collected in Table S5.

# Supplementary results and tables

## Model fit indices

This section reports fit indices for all the models tested in the present work.

The cognitive measurement models had an excellent fit to the data: comparative fit indices (CFI and TLI) were above .95, RMSEA was below .052 and SRMR below .06.

Regression models on the full sample exhibited an equally good fit: CFI and TLI indices were close to or above .95 (The worse being the age-adjusted regression model, controlling for age differences within each assessment wave: CFI = .940 and TLI = .939). Comparative fit indices decreased with restricted samples: the full regression model (including *g* and domain-specific abilities) on the higher-reliability subsample had CFI = .927 and TLI = .925. The RMSEA index value was ≤ .05 in all models. In contrast, SRMR seemed more sensitive to model complexity, with higher values for models estimating g and domain-specific abilities, and lower values for individual domain models.

**Table S1. Model fit indices**

| **Model** | **CFI** | **TLI** | **RMSEA** | **SRMR** |
| --- | --- | --- | --- | --- |
| Cognitive measurement models |  |  |  |  |
| *Bifactor* | 0.956 | 0.954 | 0.030 | 0.057 |
| *Visuospatial abilities* | 0.990 | 0.989 | 0.025 | 0.036 |
| *Verbal Memory* | 0.958 | 0.955 | 0.052 | 0.038 |
| *Processing Speed* | 0.955 | 0.952 | 0.050 | 0.055 |
| Unadjusted regression models |  |  |  |  |
| Residual 11-70 change |  |  |  |  |
| *g and domain-specific abilities* | 0.954 | 0.954 | 0.029 | 0.068 |
| *Visuospatial abilities* | 0.983 | 0.983 | 0.027 | 0.040 |
| *Verbal Memory* | 0.956 | 0.956 | 0.044 | 0.038 |
| *Processing Speed* | 0.947 | 0.948 | 0.046 | 0.057 |
| Raw 11-70 change – full sample |  |  |  |  |
| *g and domain-specific abilities* | 0.941 | 0.939 | 0.031 | 0.157 |
| Raw 11-70 change – subsample R_xx_ = .90 |  |  |  |  |
| *g and domain-specific abilities* | 0.937 | 0.936 | 0.030 | 0.136 |
| Raw 11-70 change – subsample R_xx_ = .80 |  |  |  |  |
| *g and domain-specific abilities* | 0.927 | 0.925 | 0.033 | 0.148 |
| Age-adjusted regression models |  |  |  |  |
| Residual 11-70 change |  |  |  |  |
| *g and domain-specific abilities* | 0.940 | 0.939 | 0.029 | 0.136 |

**Table S1 - continued**

| **Model** | **CFI** | **TLI** | **RMSEA** | **SRMR** |
| --- | --- | --- | --- | --- |
| NART-based regression models (unadjusted) |  |  |  |  |
| Change from age 11 to NART |  |  |  |  |
| *g and domain-specific abilities* | 0.944 | 0.943 | 0.030 | 0.129 |
| *Visuospatial abilities* | 0.984 | 0.983 | 0.022 | 0.036 |
| *Verbal Memory* | 0.946 | 0.944 | 0.042 | 0.041 |
| *Processing Speed* | 0.942 | 0.941 | 0.042 | 0.050 |
| Change from NART to age 70 |  |  |  |  |
| *g and domain-specific abilities* | 0.943 | 0.942 | 0.030 | 0.149 |
| *Visuospatial abilities* | 0.982 | 0.981 | 0.024 | 0.038 |
| *Verbal Memory* | 0.947 | 0.945 | 0.041 | 0.042 |
| *Processing Speed* | 0.942 | 0.941 | 0.042 | 0.052 |

*Note*. Models are grouped by predictor and defined by their outcome measures (in italic). In all regression models: loadings, intercepts, and covariances previously estimated in the measurement models were fixed, whereas (residual) factor variances and regression coefficients were freely estimated. Age-adjusted models covaried task scores in Waves 1 through 5 with mean-centred age in days at each assessment. Subsample Rxx = subsample showing reliable 1170 change when assuming the indicated value of test-retest reliability (i.e., .90 or .80)

## Cognitive measurement model - factor loadings

Loadings on the first-order factors (i.e., task *baseline level* and *slope*) were identical across tasks. Observed scores had an unstandardized loading of 1.00 on the *baseline level* factor. The unstandardized loading on the *slope* factor depended on the assessment wave and expressed the time in years since Wave 1: λ = 0.00; 2.98; 6.75; 9.82; 12.54 respectively for Wave 1, 2, 3, 4, and 5.

Table S2 presents the standardized loadings of task parameters on second-order general and domain-specific factors.

**Table S2. Cognitive measurement model’s standardized factor loadings**

| Observed Task | Second-order factor | | | | | | | | |
| --- | --- | --- | --- | --- | --- | --- | --- | --- | --- |
|  | Baseline level | | | |  | Slope | | | |
|  | *g* | VIS | MEM | SPE |  | *g* | VIS | MEM | SPE |
| *Matrix reasoning* | 0.787 | 0.252 |  |  |  | 0.831 | -0.063 |  |  |
| *Block design^1.2^* | 0.745 | 0.667 |  |  |  | 0.750 | -0.661 |  |  |
| *Spatial span^2^* | 0.768 | 0.049 |  |  |  | 0.957 | -0.291 |  |  |
| *Verbal paired associates^2^* | 0.441 |  | 0.647 |  |  | 0.637 |  | 0.771 |  |
| *Logical memory* | 0.518 |  | 0.616 |  |  | 0.728 |  | 0.618 |  |
| *Digit span backward* | 0.634 |  | 0.196 |  |  | 0.768 |  | 0.398 |  |
| *Symbol search^2^* | 0.822 |  |  | 0.447 |  | 0.945 |  |  | 0.326 |
| *Digit-symbol substitution* | 0.624 |  |  | 0.601 |  | 0.899 |  |  | 0.127 |
| *Inspection time* | 0.544 |  |  | 0.282 |  | 0.922 |  |  | 0.241 |
| *Four-choice RT^2^* | 0.561 |  |  | 0.491 |  | 0.938 |  |  | -0.346 |

*Note.* Loading of task parameters on general and relevant domain’s parameters. *g* = general cognitive ability, VIS = visuospatial, MEM = verbal memory, SPE = processing speed.

¹ The residual variance of the baseline level parameter was fixed at 0

^2^ The residual variance of the slope parameter was fixed at 0

## Estimating cognitive change from 11 to 70 years as a raw difference score

Assuming an MHT reliability of .90, 69.75% of the sample showed a reliable change in scores (Wave 1 N = 761). Assuming an MHT reliability of .80, 48.95% of the sample showed a reliable change in scores (Wave 1 N = 534).

In the full-data models (Table S3a), the raw difference predictor showed significant associations with g baseline level and slope. Participants exhibiting the greatest 11-70 improvement in MHT showed slower decline (β = 0.205. p = .001) in general cognitive ability. Regarding domain-specific abilities beyond g, greater 11-70 improvement corresponded to slower decline in verbal memory (β = 0.202. p = .010).

When considering only participants who exhibited reliable cognitive change from 11 to 70 years (Table S3b and S3c), the associations between age 11 to 70 change and *g* parameters resembled those described above. However, in these models, the effects on *g* slope did not survive FDR correction. Greater improvement between childhood and age 70 was significantly associated with better domain-specific verbal memory at 70 years, only in the smallest sample.

**Table S3a. Associations between raw 11-70 cognitive change and later-life trajectories of general and domain-specific^1^ cognitive abilities (full data)**

| Effect | Baseline Level | | |  | Slope | | |
| --- | --- | --- | --- | --- | --- | --- | --- |
|  | β | C.I. | *p* |  | β | C.I. | *p* |
| *g* |  |  |  |  |  |  |  |
| *11-70 Change* | **.464** | **[.39, .54]** | **.000** |  | **.205** | **[.08, .33]** | **.001** |
| *Sex* | **-.163** | **[-.22, -.10]** | **.000** |  | .094 | [.01, .18] | .027 |
| *11-70 Change × Sex* | .023 | [-.04, .08] | .446 |  | .026 | [-.06, .11] | .552 |
| *MHT 11* | **.760** | **[.69, .83]** | **.000** |  | .110 | [-.03, .25] | .112 |
| *Father social class* | .006 | [-.05, .07] | .857 |  | -.033 | [-.12, .05] | .454 |
| *Father education* | -.019 | [-.09, .05] | .575 |  | -.023 | [-.12, .08] | .644 |
| *Social class* | -.029 | [-.09, .03] | .363 |  | .032 | [-.05, .12] | .469 |
| *Education* | .016 | [-.03, .06] | .456 |  | .014 | [-.05, .07] | .647 |
| Visuospatial Ability |  |  |  |  |  |  |  |
| *11-70 Change* | -.143 | [-.26, -.02] | .019 |  | -.156 | [-.37, .06] | .152 |
| *Sex* | -.096 | [-.18, -.01] | .028 |  | .082 | [-.06, .23] | .267 |
| *11-70 Change × Sex* | .008 | [-.08, .10] | .866 |  | -.163 | [-.31, -.02] | .028 |
| *MHT 11* | -.012 | [-.15, .12] | .858 |  | **-.559** | **[-.77, -.35]** | **.000** |
| *Father social class* | -.021 | [-.11, .07] | .652 |  | .076 | [-.07, .23] | .317 |
| *Father education* | .060 | [-.04, .16] | .255 |  | -.053 | [-.23, .12] | .544 |
| *Social class* | **-.146** | **[-.23, -.06]** | **.001** |  | -.009 | [-.16, .14] | .908 |
| *Education* | .020 | [-.04, .08] | .527 |  | .004 | [-.10, .11] | .933 |
| Verbal Memory |  |  |  |  |  |  |  |
| *11-70 Change* | .047 | [-.06, .16] | .402 |  | **.202** | **[.05, .36]** | **.010** |
| *Sex* | **.345** | **[.27, .42]** | **.000** |  | .028 | [-.08, .13] | .599 |
| *11-70 Change × Sex* | -.050 | [-.13, .03] | .229 |  | .049 | [-.06, .16] | .371 |
| *MHT 11* | **.248** | **[.13, .37]** | **.000** |  | .176 | [.01, .35] | .042 |
| *Father social class* | -.044 | [-.13, .04] | .293 |  | .099 | [-.01, .20] | .066 |
| *Father education* | -.059 | [-.15, .04] | .222 |  | .057 | [-.06, .18] | .356 |
| *Social class* | .017 | [-.07, .10] | .695 |  | -.050 | [-.16, .06] | .362 |
| *Education* | .038 | [-.02, .09] | .192 |  | -.046 | [-.12, .03] | .210 |
| Processing Speed^2^ |  |  |  |  |  |  |  |
| *11-70 Change* | .013 | [-.10, .13] | .823 |  | -.252 | [-.47, -.03] | .023 |
| *Sex* | **.355** | **[.28, .43]** | **.000** |  | -.119 | [-.26, .02] | .102 |
| *11-70 Change × Sex* | -.033 | [-.12, .05] | .453 |  | .017 | [-.14, .17] | .823 |
| *MHT 11* | .019 | [-.11, .15] | .772 |  | -.178 | [-.42, .07] | .152 |
| *Father social class* | -.062 | [-.15, .02] | .161 |  | .100 | [-.05, .25] | .186 |
| *Father education* | .073 | [-.03, .17] | .159 |  | .165 | [.00, .33] | .054 |
| *Social class* | -.086 | [-.17, .00] | .054 |  | .013 | [-.14, .16] | .862 |
| *Education* | .010 | [-.05, .07] | .748 |  | -.111 | [-.21, -.01] | .030 |

*Note.* Standardized regression coefficients and p-values. 11-70 change × sex = 11-70 cognitive change × sex interaction; proportion of domain-specific slope variance beyond *g*: visuospatial 17.5%, verbal memory 37.8%, processing speed 7.5%. Bold typeface denotes FDR significant (q < .05).
^1^ Bifactor measurement model: domain-specific variance does not include variance common to all tasks (captured by *g*)
^2^ The slope of 4-choice RT task loaded negatively on the domain factor.

**Table S3b. Associations between raw 11-70 cognitive change and later-life trajectories of general and domain-specific^1^ cognitive abilities (R_xx_ = .90)**

| Effect | Baseline Level | | |  | Slope | | |
| --- | --- | --- | --- | --- | --- | --- | --- |
|  | β | C.I. | *p* |  | β | C.I. | *p* |
| *g* |  |  |  |  |  |  |  |
| *11-70 Change* | **.391** | **[.30, .48]** | **.000** |  | .191 | [.04, .34] | .012 |
| *Sex* | **-.158** | **[-.23, -.09]** | **.000** |  | .099 | [.00, .20] | .060 |
| *11-70 Change × Sex* | -.006 | [-.08, .07] | .869 |  | .055 | [-.05, .16] | .316 |
| *MHT 11* | **.742** | **[.67, .82]** | **.000** |  | .124 | [-.03, .28] | .117 |
| *Father social class* | .017 | [-.06, .09] | .656 |  | -.089 | [-.20, .02] | .102 |
| *Father education* | -.014 | [-.10, .07] | .750 |  | -.054 | [-.18, .07] | .401 |
| *Social class* | -.005 | [-.08, .07] | .888 |  | .045 | [-.06, .15] | .407 |
| *Education* | .022 | [-.03, .07] | .385 |  | .044 | [-.03, .12] | .234 |
| Visuospatial Ability |  |  |  |  |  |  |  |
| *11-70 Change* | -.158 | [-.29, -.02] | .022 |  | -.165 | [-.40, .07] | .173 |
| *Sex* | -.067 | [-.17, .04] | .206 |  | .031 | [-.14, .20] | .717 |
| *11-70 Change × Sex* | -.038 | [-.14, .07] | .468 |  | -.139 | [-.31, .03] | .104 |
| *MHT 11* | -.074 | [-.22, .07] | .319 |  | **-.454** | **[-.68, -.23]** | **.000** |
| *Father social class* | -.020 | [-.13, .09] | .710 |  | .137 | [-.03, .31] | .115 |
| *Father education* | .125 | [.00, .25] | .045 |  | -.059 | [-.26, .14] | .563 |
| *Social class* | -.164 | [-.27, -.06] | .002 |  | -.075 | [-.24, .09] | .386 |
| *Education* | -.008 | [-.08, .06] | .822 |  | -.005 | [-.12, .11] | .936 |
| Verbal Memory |  |  |  |  |  |  |  |
| *11-70 Change* | .141 | [.02, .26] | .026 |  | .035 | [-.16, .23] | .721 |
| *Sex* | **.333** | **[.25, .42]** | **.000** |  | .067 | [-.06, .20] | .309 |
| *11-70 Change × Sex* | .021 | [-.07, .12] | .655 |  | -.007 | [-.14, .13] | .921 |
| *MHT 11* | **.277** | **[.15, .41]** | **.000** |  | .077 | [-.12, .27] | .444 |
| *Father social class* | .020 | [-.08, .12] | .686 |  | .095 | [-.04, .23] | .163 |
| *Father education* | .002 | [-.11, .12] | .974 |  | .055 | [-.10, .21] | .474 |
| *Social class* | .027 | [-.07, .12] | .580 |  | -.066 | [-.20, .07] | .328 |
| *Education* | -.005 | [-.07, .06] | .891 |  | -.035 | [-.12, .05] | .435 |
| Processing Speed^2^ |  |  |  |  |  |  |  |
| *11-70 Change* | -.020 | [-.15, .11] | .764 |  | -.256 | [-.51, .00] | .052 |
| *Sex* | **.392** | **[.31, .47]** | **.000** |  | -.017 | [-.20, .17] | .857 |
| *11-70 Change × Sex* | -.097 | [-.19, .00] | .047 |  | .063 | [-.12, .25] | .511 |
| *MHT 11* | -.003 | [-.14, .13] | .970 |  | -.107 | [-.38, .17] | .444 |
| *Father social class* | -.090 | [-.19, .01] | .076 |  | .230 | [.05, .41] | .012 |
| *Father education* | .104 | [-.01, .22] | .080 |  | .159 | [-.05, .37] | .137 |
| *Social class* | -.103 | [-.20, .00] | .041 |  | .076 | [-.11, .26] | .419 |
| *Education* | .012 | [-.06, .08] | .734 |  | **-.170** | **[-.29, .05]** | **.005** |

*Note.* Standardized regression coefficients and p-values. 11-70 change × sex = 11-70 cognitive change × sex interaction; proportion of domain-specific slope variance beyond *g*: visuospatial 17.5%, verbal memory 37.8%, processing speed 7.5%. Bold typeface denotes FDR significant (q < .05).
^1^ Bifactor measurement model: domain-specific variance does not include variance common to all tasks (captured by *g*)
^2^ The slope of 4-choice RT task loaded negatively on the domain factor.

**Table S3c. Associations between raw 11-70 cognitive change and later-life trajectories of general and domain-specific^1^ cognitive abilities (R_xx_ = .80)**

| Effect | Baseline Level | | |  | Slope | | |
| --- | --- | --- | --- | --- | --- | --- | --- |
|  | β | C.I. | *p* |  | β | C.I. | *p* |
| *g* |  |  |  |  |  |  |  |
| *11-70 Change* | **.337** | **[.23, .44]** | **.000** |  | .142 | [-.04, .32] | .122 |
| *Sex* | **-.169** | **[-.25, -.08]** | **.000** |  | .115 | [-.01, .24] | .067 |
| *11-70 Change × Sex* | -.009 | [-.10, .08] | .844 |  | .089 | [-.04, .22] | .174 |
| *MHT 11* | **.701** | **[.61, .79]** | **.000** |  | .100 | [-.08, .28] | .271 |
| *Father social class* | .044 | [-.04, .13] | .321 |  | -.088 | [-.21, .04] | .173 |
| *Father education* | -.033 | [-.14, .07] | .524 |  | -.143 | [-.30, .01] | .068 |
| *Social class* | -.011 | [-.10, .08] | .798 |  | .092 | [-.03, .22] | .140 |
| *Education* | .039 | [-.02, .10] | .188 |  | .081 | [-.01, .17] | .067 |
| Visuospatial Ability |  |  |  |  |  |  |  |
| *11-70 Change* | -.154 | [-.31, .00] | .054 |  | -.171 | [-.46, .12] | .255 |
| *Sex* | -.095 | [-.22, .03] | .127 |  | .016 | [-.18, .22] | .873 |
| *11-70 Change × Sex* | .002 | [-.12, .13] | .974 |  | -.222 | [-.42, -.02] | .030 |
| *MHT 11* | -.067 | [-.23, .10] | .430 |  | **-.430** | **[-.70, -.16]** | **.002** |
| *Father social class* | -.050 | [-.18, .08] | .434 |  | .175 | [-.02, .37] | .084 |
| *Father education* | .077 | [-.07, .23] | .316 |  | -.048 | [-.30, .20] | .706 |
| *Social class* | **-.179** | **[-.30, -.06]** | **.004** |  | -.123 | [-.32, .07] | .216 |
| *Education* | .022 | [-.06, .11] | .614 |  | -.012 | [-.15, .13] | .864 |
| Verbal Memory |  |  |  |  |  |  |  |
| *11-70 Change* | **.208** | **[.07, .35]** | **.004** |  | -.037 | [-.27, .19] | .749 |
| *Sex* | **.367** | **[.27, .46]** | **.000** |  | .082 | [-.07, .23] | .287 |
| *11-70 Change × Sex* | -.017 | [-.13, .10] | .773 |  | -.028 | [-.19, .13] | .728 |
| *MHT 11* | **.298** | **[.16, .44]** | **.000** |  | .047 | [-.18, .27] | .678 |
| *Father social class* | -.015 | [-.13, .10] | .799 |  | .108 | [-.05, .26] | .169 |
| *Father education* | .074 | [-.06, .21] | .292 |  | .154 | [-.03, .33] | .095 |
| *Social class* | .072 | [-.04, .18] | .204 |  | -.142 | [-.29, .01] | .059 |
| *Education* | -.038 | [-.11, .04] | .334 |  | -.064 | [-.17, .04] | .222 |
| Processing Speed^2^ |  |  |  |  |  |  |  |
| *11-70 Change* | .033 | [-.11, .18] | .657 |  | -.096 | [-.42, .23] | .565 |
| *Sex* | **.360** | **[.26, .46]** | **.000** |  | .032 | [-.19, .25] | .771 |
| *11-70 Change × Sex* | -.099 | [-.22, .02] | .094 |  | .071 | [-.16, .30] | .539 |
| *MHT 11* | .013 | [-.14, .17] | .864 |  | .125 | [-.20, .45] | .447 |
| *Father social class* | -.109 | [-.23, .01] | .065 |  | .267 | [.06, .47] | .011 |
| *Father education* | .129 | [-.01, .27] | .071 |  | .122 | [-.14, .39] | .363 |
| *Social class* | -.125 | [-.24, -.01] | .034 |  | .121 | [-.09, .34] | .271 |
| *Education* | .006 | [-.07, .09] | .883 |  | -.174 | [-.31, -.03] | .015 |

*Note.* Standardized regression coefficients and p-values. 11-70 change × sex = 11-70 cognitive change × sex interaction; proportion of domain-specific slope variance beyond *g*: visuospatial 17.5%, verbal memory 37.8%, processing speed 7.5%. Bold typeface denotes FDR significant (q < .05).
^1^ Bifactor measurement model: domain-specific variance does not include variance common to all tasks (captured by *g*)
^2^ The slope of 4-choice RT task loaded negatively on the domain factor.

## Age-adjusted models

**Table S4.** **Associations between 11-70 cognitive change and later-life trajectories of general and domain-specific^1^ cognitive abilities, controlling for age differences at the time of assessments**

| Effect | Baseline Level | | |  | Slope | | |
| --- | --- | --- | --- | --- | --- | --- | --- |
|  | β | C.I. | *p* |  | β | C.I. | *p* |
| *g* |  |  |  |  |  |  |  |
| *11-70 Change* | **.350** | **[.29, .41]** | **.000** |  | **.152** | **[.06, .25]** | **.002** |
| *Sex* | **-.164** | **[-.22, -.11]** | **.000** |  | .087 | [.00, .17] | .040 |
| *11-70 Change × Sex* | .062 | [.00, .12] | .046 |  | -.013 | [-.11, .08] | .790 |
| *MHT 11* | **.452** | **[.39, .51]** | **.000** |  | -.019 | [-.12, .08] | .701 |
| *Father social class* | .005 | [-.06, .07] | .879 |  | -.034 | [-.12, .05] | .442 |
| *Father education* | -.021 | [-.09, .05] | .542 |  | -.017 | [-.12, .08] | .737 |
| *Social class* | -.031 | [-.09, .03] | .325 |  | .026 | [-.06, .11] | .561 |
| *Education* | .018 | [-.02, .06] | .397 |  | .012 | [-.05, .07] | .678 |
| Visuospatial Ability |  |  |  |  |  |  |  |
| *11-70 Change* | -.098 | [-.18, -.01] | .024 |  | -.129 | [-.29, .03] | .111 |
| *Sex* | -.011 | [-.10, .08] | .807 |  | .102 | [-.04, .24] | .161 |
| *11-70 Change × Sex* | .084 | [-.02, .18] | .099 |  | **-.243** | **[-.39, -.09]** | **.001** |
| *MHT 11* | -.021 | [-.11, .07] | .645 |  | **-.457** | **[-.60, -.32]** | **.000** |
| *Father social class* | .058 | [-.04, .16] | .270 |  | .080 | [-.07, .23] | .282 |
| *Father education* | **-.144** | **[-.23, -.06]** | **.001** |  | -.043 | [-.21, .13] | .620 |
| *Social class* | .020 | [-.04, .08] | .527 |  | -.010 | [-.16, .14] | .894 |
| *Education* | -.098 | [-.18, -.01] | .024 |  | -.002 | [-.10, .10] | .976 |
| Verbal Memory |  |  |  |  |  |  |  |
| *11-70 Change* | .044 | [-.04, .13] | .298 |  | .137 | [.02, .25] | .022 |
| *Sex* | **.324** | **[.25, .39]** | **.000** |  | .037 | [-.07, .14] | .488 |
| *11-70 Change × Sex* | -.070 | [-.15, .01] | .087 |  | -.010 | [-.13, .11] | .869 |
| *MHT 11* | **.230** | **[.14, .32]** | **.000** |  | .029 | [-.09, .15] | .644 |
| *Father social class* | -.038 | [-.12, .04] | .353 |  | .087 | [-.02, .19] | .102 |
| *Father education* | -.067 | [-.16, .03] | .157 |  | .066 | [-.05, .19] | .275 |
| *Social class* | .020 | [-.06, .10] | .637 |  | -.048 | [-.15, .06] | .383 |
| *Education* | .038 | [-.02, .09] | .173 |  | -.048 | [-.12, .02] | .190 |
| Processing Speed^2^ |  |  |  |  |  |  |  |
| *11-70 Change* | .005 | [-.08, .09] | .903 |  | -.162 | [-.33, .01] | .059 |
| *Sex* | **.351** | **[.28, .42]** | **.000** |  | -.142 | [-.28, .00] | .051 |
| *11-70 Change × Sex* | -.107 | [-.19, -.02] | .014 |  | -.015 | [-.18, .15] | .861 |
| *MHT 11* | .017 | [-.08, .11] | .729 |  | .012 | [-.16, .19] | .892 |
| *Father social class* | -.061 | [-.15, .02] | .163 |  | .093 | [-.05, .24] | .216 |
| *Father education* | .072 | [-.03, .17] | .159 |  | .159 | [-.01, .33] | .063 |
| *Social class* | -.082 | [-.17, .00] | .064 |  | .009 | [-.14, .16] | .906 |
| *Education* | .008 | [-.05, .07] | .788 |  | -.106 | [-.21, -.01] | .038 |

*Note.* Standardized coefficients and p-values. *11-70 change* × sex = cognitive change from 11 to 70 × sex interaction; proportion of variance captured by domain-specific factors beyond *g*: visuospatial 17.1%, verbal memory 38.7%, processing speed 7.9%
^1^ Domain-specific variance does not include general variance common to all tasks (captured by *g*)
^2^ 4-choice RT task slope loaded negatively on the domain factor.

Bold typeface denotes p-values that survived FDR correction (q < 0.05).

## Individual domain models

**Table S5. Associations between cognitive change from 11 to 70 years and later-life trajectories of individual cognitive domains**

| Effect | Baseline Level | | |  | Slope | | |
| --- | --- | --- | --- | --- | --- | --- | --- |
|  | β | C.I. | *p* |  | β | C.I. | *p* |
| Residual 11-70 change |  |  |  |  |  |  |  |
| Visuospatial Ability |  |  |  |  |  |  |  |
| *11-70 Change* | **.391** | **[.34, .44]** | **.000** |  | -.050 | [-.19, .09] | .491 |
| *Sex* | **-.212** | **[-.26, -.16]** | **.000** |  | .153 | [.03, .28] | .016 |
| *11-70 Change × Sex* | **.078** | **[.03, .13]** | **.003** |  | **-.233** | **[-.37, -.09]** | **.001** |
| *MHT 11* | **.571** | **[.52, .62]** | **.000** |  | **-.317** | **[-.47, -.17]** | **.000** |
| *Father social class* | .001 | [-.05, .05] | .972 |  | -.040 | [-.17, .09] | .542 |
| *Father education* | .019 | [-.04, .08] | .532 |  | -.103 | [-.25, .04] | .172 |
| *Social class* | **-.097** | **[-.15, -.04]** | **.000** |  | -.053 | [-.18, .07] | .417 |
| *Education* | .019 | [-.02, .05] | .291 |  | .071 | [-.02, .16] | .119 |
| Verbal Memory |  |  |  |  |  |  |  |
| *11-70 Change* | **.339** | **[.28, .40]** | **.000** |  | **.210** | **[.11, .31]** | **.000** |
| *Sex* | **.136** | **[.08, .19]** | **.000** |  | .085 | [.00, .17] | .055 |
| *11-70 Change × Sex* | -.007 | [-.07, .05] | .824 |  | .007 | [-.09, .10] | .893 |
| *MHT 11* | **.609** | **[.55, .67]** | **.000** |  | .004 | [-.12, .13] | .950 |
| *Father social class* | -.022 | [-.08, .04] | .477 |  | .043 | [-.05, .13] | .340 |
| *Father education* | -.075 | [-.15, .00] | .039 |  | .048 | [-.05, .15] | .354 |
| *Social class* | -.006 | [-.07, .06] | .851 |  | -.010 | [-.10, .08] | .823 |
| *Education* | .045 | [.00, .09] | .037 |  | -.034 | [-.09, .03] | .272 |
| Processing Speed |  |  |  |  |  |  |  |
| *11-70 Change* | **.380** | **[.33, .43]** | **.000** |  | **.143** | **[.04, .24]** | **.005** |
| *Sex* | .043 | [-.01, .10] | .115 |  | **.117** | **[.03, .20]** | **.009** |
| *11-70 Change × Sex* | -.015 | [-.07, .04] | .594 |  | .004 | [-.10, .10] | .938 |
| *MHT 11* | **.480** | **[.43, .54]** | **.000** |  | -.005 | [-.15, .14] | .945 |
| *Father social class* | -.032 | [-.09, .02] | .259 |  | -.014 | [-.11, .08] | .761 |
| *Father education* | .027 | [-.04, .09] | .416 |  | -.004 | [-.11, .10] | .946 |
| *Social class* | **-.081** | **[-.14, -.03]** | **.004** |  | .034 | [-.06, .12] | .468 |
| *Education* | .020 | [-.02, .06] | .301 |  | -.003 | [-.07, .06] | .934 |

| Effect | Baseline Level | | |  | Slope | | |
| --- | --- | --- | --- | --- | --- | --- | --- |
|  | β | C.I. | *p* |  | β | C.I. | *p* |
| Change from age 11 MHT to NART |  |  |  |  |  |  |  |
| Visuospatial Ability |  |  |  |  |  |  |  |
| *11-NART Change* | **.145** | **[.09, .20]** | **.000** |  | .002 | [-.14, .14] | .974 |
| *Sex* | **-.258** | **[-.31, -.21]** | **.000** |  | .144 | [.02, .27] | .023 |
| *11-NART Change × Sex* | .059 | [.00, .11] | .037 |  | **-.168** | **[-.30, -.04]** | **.010** |
| *MHT 11* | **.557** | **[.50, .61]** | **.000** |  | **-.316** | **[-.47, -.17]** | **.000** |
| *Father social class* | -.005 | [-.06, .05] | .858 |  | -.032 | [-.16, .10] | .626 |
| *Father education* | -.014 | [-.08, .05] | .685 |  | -.090 | [-.24, .06] | .233 |
| *Social class* | **-.117** | **[-.17, -.06]** | **.000** |  | -.038 | [-.17, .09] | .576 |
| *Education* | .041 | [.00, .08] | .041 |  | .062 | [-.03, .15] | .182 |
| Verbal Memory |  |  |  |  |  |  |  |
| *11-NART Change* | **.292** | **[.23, .35]** | **.000** |  | .098 | [.00, .19] | .046 |
| *Sex* | **.096** | **[.04, .15]** | **.001** |  | .065 | [-.02, .15] | .146 |
| *11-NART Change × Sex* | .025 | [-.03, .09] | .411 |  | -.006 | [-.10, .09] | .903 |
| *MHT 11* | .634 | [.57, .69] | .000 |  | -.011 | [-.14, .11] | .858 |
| *Father social class* | .000 | [-.06, .06] | .993 |  | .046 | [-.04, .14] | .314 |
| *Father education* | **-.098** | **[-.17, -.03]** | **.008** |  | .036 | [-.07, .14] | .487 |
| *Social class* | .017 | [-.05, .08] | .599 |  | -.012 | [-.11, .08] | .804 |
| *Education* | .042 | [.00, .09] | .061 |  | -.028 | [-.09, .03] | .376 |
| Processing Speed |  |  |  |  |  |  |  |
| *11- NART Change* | **.096** | **[.03, .16]** | **.003** |  | .097 | [.00, .19] | .048 |
| *Sex* | .003 | [-.05, .06] | .914 |  | .103 | [.02, .19] | .022 |
| *11-NART Change × Sex* | .005 | [-.05, .06] | .864 |  | -.017 | [-.11, .07] | .718 |
| *MHT 11* | **.461** | **[.40, .52]** | **.000** |  | -.006 | [-.15, .14] | .933 |
| *Father social class* | -.046 | [-.11, .01] | .137 |  | -.007 | [-.10, .09] | .877 |
| *Father education* | -.008 | [-.08, .06] | .819 |  | -.007 | [-.11, .10] | .890 |
| *Social class* | **-.109** | **[-.17, -.05]** | **.000** |  | .041 | [-.05, .13] | .391 |
| *Education* | .049 | [.01, .09] | .023 |  | -.005 | [-.07, .06] | .883 |
| Change from NART to age 70 MHT |  |  |  |  |  |  |  |
| Visuospatial Ability |  |  |  |  |  |  |  |
| *NART-70 Change* | **.324** | **[.27, .38]** | **.000** |  | -.043 | [-.19, .10] | .564 |
| *Sex* | **-.219** | **[-.27, -.17]** | **.000** |  | .143 | [.02, .27] | .027 |
| *NART-70 Change × Sex* | .056 | [.00, .11] | .034 |  | -.079 | [-.22, .06] | .262 |
| *MHT 11* | **.429** | **[.37, .49]** | **.000** |  | **-.296** | **[-.45, -.14]** | **.000** |
| *Father social class* | -.039 | [-.09, .01] | .149 |  | -.033 | [-.16, .10] | .613 |
| *Father education* | -.001 | [-.06, .06] | .964 |  | -.101 | [-.25, .05] | .180 |
| *Social class* | **-.154** | **[-.21, -.10]** | **.000** |  | -.046 | [-.17, .08] | .482 |
| *Education* | **.058** | **[.02, .09]** | **.002** |  | .068 | [-.02, .16] | .134 |
| Verbal Memory |  |  |  |  |  |  |  |
| *NART-70 Change* | **.179** | **[.11, .24]** | **.000** |  | **.151** | **[.05, .25]** | **.003** |
| *Sex* | **.121** | **[.06, .18]** | **.000** |  | .082 | [-.01, .17] | .066 |
| *NART-70 Change × Sex* | .006 | [-.06, .07] | .847 |  | -.003 | [-.10, .09] | .950 |
| *MHT 11* | **.520** | **[.45, .59]** | **.000** |  | -.066 | [-.19, .06] | .315 |
| *Father social class* | -.055 | [-.12, .01] | .086 |  | .026 | [-.06, .12] | .559 |
| *Father education* | **-.104** | **[-.18, -.03]** | **.005** |  | .038 | [-.06, .14] | .460 |
| *Social class* | -.053 | [-.12, .01] | .095 |  | -.040 | [-.13, .05] | .378 |
| *Education* | **.083** | **[.04, .13]** | **.000** |  | -.016 | [-.08, .04] | .598 |
| Processing Speed |  |  |  |  |  |  |  |
| *NART-70 Change* | **.354** | **[.30, .41]** | **.000** |  | .059 | [-.04, .16] | .262 |
| *Sex* | .043 | [-.01, .10] | .119 |  | .112 | [.02, .20] | .014 |
| *NART-70 Change × Sex* | .014 | [-.04, .07] | .623 |  | -.001 | [-.10, .10] | .983 |
| *MHT 11* | **.330** | **[.27, .39]** | **.000** |  | -.044 | [-.19, .10] | .566 |
| *Father social class* | **-.073** | **[-.13, -.02]** | **.010** |  | -.024 | [-.12, .07] | .611 |
| *Father education* | .008 | [-.06, .07] | .823 |  | -.012 | [-.12, .09] | .825 |
| *Social class* | **-.139** | **[-.19, -.08]** | **.000** |  | .015 | [-.08, .11] | .753 |
| *Education* | **.059** | **[.02, .10]** | **.002** |  | .010 | [-.05, .07] | .761 |

*Note.* Models are grouped by predictor. Standardized regression coefficients and p-values are reported. *11-70 change* × sex = cognitive change from 11 to 70 × sex interaction.

Bold typeface denotes p-values that survived FDR correction (q < 0.05).
